# The shortened disabilities of the arm, shoulder and hand questionnaire (*Quick*DASH): validity and reliability based on responses within the full-length DASH

**DOI:** 10.1186/1471-2474-7-44

**Published:** 2006-05-18

**Authors:** Christina Gummesson, Michael M Ward, Isam Atroshi

**Affiliations:** 1 National Institute of Arthritis and Musculoskeletal and Skin Diseases, NIH, Bethesda, Maryland 20892, USA; 2 Department of Health Sciences, Division of Physiotherapy, Lund University, Lund, SE 22185, Sweden; 3 Department of Orthopedics Hässleholm-Kristianstad, Hässleholm Hospital, Hässleholm SE 28125, Sweden

## Abstract

**Background:**

The 30-item disabilities of the arm, shoulder and hand (DASH) questionnaire is increasingly used in clinical research involving upper extremity musculoskeletal disorders. From the original DASH a shorter version, the 11-item *Quick*DASH, has been developed. Little is known about the discriminant ability of score changes for the *Quick*DASH compared to the DASH. The aim of this study was to assess the performance of the *Quick*DASH and its cross-sectional and longitudinal validity and reliability.

**Methods:**

The study was based on extracting *Quick*DASH item responses from the responses to the full-length DASH questionnaire completed by 105 patients with a variety of upper extremity disorders before surgery and at follow-up 6 to 21 months after surgery. The DASH and *Quick*DASH scores were compared for the whole population and for different diagnostic groups. For longitudinal construct validity the effect size and standardized response mean were calculated. Analyses with ROC curves were performed to compare the ability of the DASH and *Quick*DASH to discriminate among patients classified according to the magnitude of self-rated improvement. Cross-sectional and test-retest reliability was assessed.

**Results:**

The mean DASH score was 34 (SD 22) and the mean *Quick*DASH score was 39 (SD 24) at baseline. For the different diagnostic groups the mean and median *Quick*DASH scores were higher than the corresponding DASH scores. For the whole population, the mean difference between the *Quick*DASH and DASH baseline scores was 4.2 (95% CI 3.2–5.3), follow-up scores was 2.6 (1.7–3.4), and change scores was 1.7 (0.6–2.8).

The overall effect size and standardized response mean measured with the DASH and the *Quick*DASH were similar. In the ROC analysis of change scores among patients who rated their arm status as somewhat or much better and those who rated it as unchanged the difference in the area under the ROC curve for the DASH and *Quick*DASH was 0.01 (95% CI -0.05–0.07) indicating similar discriminant ability.

Cross-sectional and test-retest reliability of the DASH and *Quick*DASH were similar.

**Conclusion:**

The results indicate that the *Quick*DASH can be used instead of the DASH with similar precision in upper extremity disorders.

## Background

Patient-reported outcome measures have become an important part of the assessments used in clinical studies. One of the outcome measures intended for upper extremity disorders is the 30-item disabilities of the arm, shoulder and hand (DASH) questionnaire, which has been assessed regarding reliability, cross-sectional validity and longitudinal validity in a variety of arm disorders [1-3]. The use of the DASH has been growing rapidly in clinical trials and other studies of upper extremity disorders and it is now available in several languages [4].

From the original DASH questionnaire a shorter version, named the *Quick*DASH, has been developed using what was called a "concept-retention" approach [5]. The *Quick*DASH consists of 11 items from the original 30-item DASH. The *Quick*DASH may be more appealing to use than the DASH because a shorter questionnaire is associated with less burden on the responder as well as less administrative burden. To date, data regarding the development process and various aspects of reliability and validity have been published only for the English version of the *Quick*DASH [5]. It is important that translated versions of shortened questionnaires also are subjected to an appropriate validation process. Furthermore, little is known about how the *Quick*DASH scores can be interpreted in comparison to the DASH scores or which version is more favorable with respect to precision of the scoring.

To determine whether a shortened questionnaire may be used to replace an existing full-length questionnaire, several assessments can be performed to show that the short version should be measuring what the original version is measuring. Different aspects of cross-sectional validity can be compared [6]. Further, longitudinal construct validity, which concerns the measure's ability to detect a true change in health status and its precision in detecting changes of different magnitudes (also referred to as responsiveness or sensitivity to change) needs to be addressed to determine the clinical usefulness of the short version [7-9].

The purpose of this study was to evaluate the performance of the 11-item *Quick*DASH in comparison to the full-length 30-item DASH regarding different aspects of validity and reliability. The data for the *Quick*DASH were extracted from the full-length DASH.

## Methods

### Design

This study was designed as a reanalysis of collected data for the 30-item DASH questionnaire, from which scores for both the DASH and the *Quick*DASH were calculated. The data collection process for the assessment of the longitudinal construct validity of the DASH has been described previously [10]. The study was conducted in agreement with the local ethical guidelines for clinical studies and informed consent was obtained from the participants.

### Questionnaire

The DASH questionnaire mainly consists of a 30-item disability/symptom scale. The two optional scales of the DASH (sport/music and work) were not part of the study. Each item in the disability/symptom scale has 5 response options. If at least 27 of the 30 items are completed a scale score, ranging from 0 (no disability) to 100 (most severe disability), can be calculated.

From the full-length DASH the 11 items that constitute the *Quick*DASH were extracted. To calculate a *Quick*DASH score at least 10 of the 11 items must be completed. Similar to the DASH, each item has 5 response options and, from the item scores, scale scores are calculated, ranging from 0 (no disability) to 100 (most severe disability).

The follow-up questionnaire included an item inquiring about change in the status of the arm as compared to its status before surgery. The item had 5 response options; much better, somewhat better, unchanged, somewhat worse, much worse. This item was accidentally missing in the initially mailed questionnaires and was therefore only completed by the last 83 participants, 82 of whom had *Quick*DASH scores and could be included in the present analysis.

### Setting and participants

From an orthopedic department 109 of 118 consecutive patients with upper extremity disorders who fulfilled the eligibility criteria (scheduled for elective surgery, 18 years or older, symptom duration of at least 2 months, able to answer questionnaires) responded to the Swedish version of the DASH before surgery and at the follow-up evaluation. The follow-up was done at 6 to 21 (mean 12) months after surgery.

Of the 109 responders, 105 had responded to at least 10 of the 11 items used in the *Quick*DASH and were included in the analysis. The mean age of the 105 participants was 52 (range 18–83) years; 60 (57%) were women and 45 were men.

### Analysis

The baseline, follow-up and change scores for the DASH and the *Quick*DASH were calculated for the whole population and for specific diagnostic groups.

To study the longitudinal construct validity the effect size (mean change score divided by the standard deviation of the baseline scores) and the standardized response mean (mean change score divided by the standard deviation of the change scores) for the DASH and *Quick*DASH were calculated.

To compare the performance of the DASH and the *Quick*DASH in discriminating among patients who differed in the degree of arm-related disability, receiver operating characteristic (ROC) curves were constructed using change scores (baseline to follow-up) as the test variable and patients' responses to the global item concerning perceived change in arm status after surgery as the dichotomized classifying variable; the difference in the areas under the ROC curves for the two questionnaire versions was calculated [11,12]. In the first ROC analysis the DASH and *Quick*DASH were compared with regard to their ability to discriminate the patients who rated their arm status as "much better" or "somewhat better" (combined into one group) from those who rated it as "unchanged". In the second analysis the ability to discriminate the "much better" group from the "somewhat better" group was compared. The difference in the areas under the ROC curves indicates the magnitude of the difference in the discriminant ability of the two measures. The number of patients who had reported worsening was too small to perform an analysis comparing the ability of the 2 measures to detect deterioration.

To assess reliability the Cronbach alpha coefficient was calculated for the baseline and follow-up item responses. Agreement between the *Quick*DASH and the full-length DASH was assessed with the intraclass correlation coefficient (ICC) using the 2-way mixed and absolute agreement model [13]. The difference between the DASH scores and the *Quick*DASH scores was assessed with the paired-samples t-test. Because the *Quick*DASH responses were extracted from the full-length DASH some degree of correlation between part of the questionnaire and the whole is expected. To explore the possible effect of this factor we created two hypothetical 11-item short-forms by computer-generated random selection from the 30 items of the full-length DASH. These random 11-item short-forms were analyzed with regard to reliability in a similar fashion as done with the *Quick*DASH.

Test-retest reliability was studied in a subgroup of 30 patients (14 women) with a mean age of 54 (range 27–79) years, who had completed the full-length DASH on two occasions prior to surgery with a median interval of 5 (range 5–17) days [14]. The scores for the DASH, *Quick*DASH and the random short-forms from both response times were calculated. The ICC (2-way mixed, absolute agreement) and the paired-samples t-test were used for this analysis.

## Results

### Cross-sectional validity

**Figure 1:**
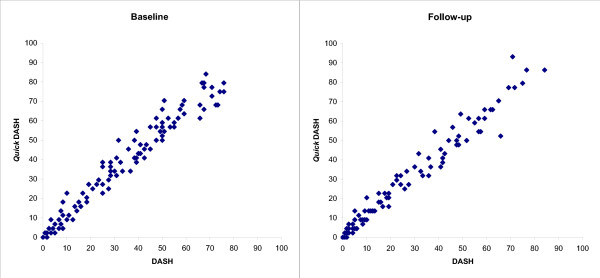
Scatter plot of the *Quick*DASH and DASH scores at baseline and follow-up.

The baseline mean DASH score was 34 (SD 22) and the mean *Quick*DASH score was 39 (SD 24) (Table 1). A best possible score of zero (ceiling) at baseline was recorded for the *Quick*DASH in 3 patients (2.9%) and for the DASH in 1 patient (1%) and a score of less than 10 was found in 19 patients (18%) and 20 patients (19%), respectively (Figure 1). At follow-up, 12 patients (14%) had a best possible *Quick*DASH and 10 (9.5%) a best possible DASH score. No patient had a score exceeding 90 at any evaluation except for 1 patient who had a *Quick*DASH score of 93 at follow-up.

The mean difference between the *Quick*DASH and the DASH scores at baseline was 4.2 (SD 5.4) and the mean difference at follow-up was 2.6 (SD 4.6). The mean difference between the *Quick*DASH and DASH change scores was 1.7 (SD 5.8; 95% CI 0.6–2.8).

For the different diagnostic groups the mean and median *Quick*DASH scores were higher than the corresponding DASH scores by up to 5 points in most groups (Table 2). Among patients with shoulder disorders the mean DASH score was 44 (SD 15) and the mean *Quick*DASH score was 49 (SD 18); the difference among patients with CTS was even larger.

### Longitudinal construct validity

When assessing the magnitude of change from baseline to follow-up the overall effect size and standardized response mean measured with the DASH and the *Quick*DASH were similar (Table 1). Among the 24 patients with shoulder disorders treated with arthroscopic acromioplasty, the effect size measured with the DASH and *Quick*DASH was 0.79 and 0.74, and the standardized response mean was 0.45 and 0.46, respectively. Among the 19 patients with carpal tunnel syndrome, the effect size of open carpal tunnel release surgery measured with the DASH and *Quick*DASH was 0.66 and 0.89 and the standardized response mean was 0.98 and 1.05, respectively.

In the ROC analysis of the change scores for the patients who rated their arm status after surgery as better (including "much better" and "somewhat better") and those who rated it as "unchanged", the difference in the area under the ROC curves for the DASH and *Quick*DASH was 0.01 (95% CI -0.05–0.07), indicating no difference in their ability to discriminate between the 2 groups (Table 3). In the ROC analysis comparing the ability to discriminate the "much better" group from the "somewhat better" group, the difference in the area under the ROC curves for the DASH and the *Quick*DASH was 0.03 (95% CI -0.03–0.09).

### Reliability

#### *Quick*DASH

In the assessment of cross-sectional reliability among the 105 responders, the alpha coefficient for the scores exceeded 0.90 and the corrected item-total correlations (ITC) exceeded 0.62, except for 1 item with ITC of 0.42 at baseline (Table 4). The ICC values for the agreement between the *Quick*DASH and the DASH scores were high, exceeding 0.90 at baseline and follow-up.

In the analysis of test-retest reliability, the ICC for the *Quick*DASH scores on the 2 response times was high and the mean difference between the *Quick*DASH scores on the first and second response time was almost zero and the 95% confidence interval was within 4 points in each direction.

#### Random 11-item forms

The first short-form included 11 randomly selected items from the full-length DASH (items 1, 3, 5, 8, 10, 11, 12, 19, 20, 24, 27). The mean score was 38 (SD 24) at baseline and 28 (SD 26) at follow-up. For the second short-form (items 3, 7, 11, 12, 13, 15, 22, 25, 26, 27, 28), the mean score was 36 (SD 23) at baseline and 25 (SD 24) at follow-up. The reliability coefficients and agreement with the DASH were high and similar to those for the *Quick*DASH (Table 4).

## Discussion

The aim of this study was to compare the performance of the 11-item *Quick*DASH with that of the 30-item DASH, with the *Quick*DASH scores extracted from the responses to the full-length DASH. The results indicate that the DASH can be replaced by the shorter *Quick*DASH. The magnitude of the differences between the DASH and the *Quick*DASH scores found in this study implies that the same questionnaire should be used in longitudinal studies because the score differences between the questionnaires may inflate small random differences and make them reach the level of an important change.

In all analyses the *Quick*DASH scores were slightly higher than the corresponding DASH scores, which may be an advantage for the *Quick*DASH as this allows for larger improvement to occur, provided that the scores considered as "normal" are equal. Among the different diagnostic groups the *Quick*DASH mean scores were higher; in fact this difference was more pronounced among patients with greater disability, such as those with shoulder disorder, than patients with little disability, such as those with wrist ganglion (Table 2). This suggested that the *Quick*DASH potentially had better precision in detecting different degrees of disability. To further assess possible differences in the two measures' ability to detect improvement, ROC curves were studied. In all analyses, the confidence intervals for the difference contained null, indicating that no differences were found between the DASH and the *Quick*DASH in their ability to discriminate among groups that differed in the degree of self-rated improvement in arm status after surgery.

In the study assessing the English-version *Quick*DASH the standardized response mean, calculated for the total population of 171 patients with various disorders, was 0.78 for the DASH and 0.79 for the *Quick*DASH [5]. In our study the standardized response mean for the DASH and *Quick*DASH also were similar, with values of 0.61 and 0.63, respectively. The mean scores for the DASH and *Quick*DASH in different diagnostic groups were more similar in the study of the English-version *Quick*DASH than in our study. However, limited data was available and the score distributions for the groups were not shown making comparisons difficult.

In this study, as in the study that reported the development and validation of the English version [5], the *Quick*DASH scores were computed from the full-length DASH responses. It is not known if patients' responses to the 11 items would have differed if only the *Quick*DASH were administered. In a study of the performance of three SF-36 scales (physical functioning, bodily pain and general health perceptions) no significant differences were found when the scales were administered independently compared to when they were administered within the full 8-scale questionnaire [15]. However, these were full scales and not selected items as is the case with the *Quick*DASH. The results of the present study, based on *Quick*DASH responses extracted from the full-length DASH, are promising but further assessment of the short version administered to different patient groups would be useful. Because of the small number of patients in certain diagnostic groups as well as the small number of patients with unchanged or worsened self-rated arm status the results involving these groups may need to be interpreted with caution.

The reliability of the *Quick*DASH was good. However, the 2 randomly constructed 11-item forms also had similarly good reliability and agreement with the DASH. The 2 random short forms showed higher scores than the DASH at baseline and follow-up, which also was found with the *Quick*DASH. Although the differences were statistically significant, their magnitude may not be considered as clinically important. The findings may suggest that the 30-item full DASH may contain redundant items and that fewer items would be sufficient for assessing disability with the same degree of reliability and validity. It might be argued that the random short forms may not cover all relevant domains. However, the results of the DASH or *Quick*DASH are usually not presented as a number of separate components or domains because they are not validated as such. Moreover, the DASH and *Quick*DASH are predominantly composed of activity items that measure physical disability leaving little impact for the non-activity items. Because the item responses were extracted from the responses to the full-length DASH it may not be possible to compare with certainty the individual performance of the *Quick*DASH as compared to other possible short forms of the DASH.

In this study all participants underwent surgery, an intervention that often results in large score change. The effect size and standardized response mean measured with DASH and *Quick*DASH in populations treated with surgery may be larger than those measured after other interventions. However, the overall effect size in this population was moderate probably because the different diagnostic groups had large variation in the degree of baseline disability with some groups having low scores before treatment allowing only small score improvement.  The results support the use of the *Quick*DASH even in the assessment of interventions expected to have smaller effect size.

The findings of this study are primarily related to the validity and reliability of the Swedish version of the *Quick*DASH (available online [4]). Although many aspects also may apply to *Quick*DASH versions that are derived from other translated full-length versions with established validity and reliability, other language versions would still require appropriate assessment.

## Conclusion

The results of this study indicate that the *Quick*DASH can be used instead of the DASH to measure disability/symptom severity with similar precision in a variety of arm disorders.

## Competing interests

The author(s) declare that they have no competing interests.

## Authors' contributions

CG and IA participated in the design of the study, data collection and analysis, and writing of this manuscript. MW participated in the analysis and writing of this manuscript. All authors read and approved the final manuscript.

## Pre-publication history

The pre-publication history for this paper can be accessed here:



## Figures and Tables

**Table 1:** The DASH and *Quick*DASH scores at baseline and follow-up for the 105 responders.

	**Baseline**		**Follow-up**		**Difference**			
	Mean (SD)	Median (IQR)	Mean (SD)	Median (IQR)	Mean (SD)	Median (IQR)	Effect Size	Standardized Response Mean
	
DASH	35 (22)	33 (15–50)	24 (23)	16 (4–42)	10 (17)	8 (1–21)	0.46	0.61
								
*Quick*DASH	39 (24)	39 17–59)	27 (25)	18 (5–47)	12 (19)	9 (0–27)	0.50	0.63

The scores for both scales range from 0 (no disability) to 100 (most severe disability).

IQR, interquartile range

**Table 2:** The DASH and *Quick*DASH scores for different diagnostic groups among the 105 responders.

		**Baseline**		**Follow-up**	
		Mean (SD)	Median	Mean (SD)	Median
		
Shoulder disorder (n = 27)	DASH	44 (15)	48	33 (24)	33
	*Quick*DASH	49 (18)	50	37 (25)	34
					
Carpal tunnel syndrome (n = 19)	DASH	41 (20)	40	28 (26)	15
	*Quick*DASH	49 (20)	46	31 (29)	23
					
Dupuytren's disease (n = 13)	DASH	19 (23)	8	15 (23)	5
	*Quick*DASH	22 (27)	9	17 (23)	7
					
Ganglion (n = 7)	DASH	15 (23)	8	11 (15)	5
	*Quick*DASH	16 (22)	14	12 (14)	9
					
Trapeziometacarpal arthritis (n = 6)	DASH	48 (17)	48	24 (22)	25
	*Quick*DASH	51 (15)	52	27 (24)	30
					
Tenosynovitis (n = 9)	DASH	35 (17)	30	20 (19)	8
	*Quick*DASH	39 (17)	34	23 (22)	9
					
Other (n = 24)	DASH	29 (24)	24	21 (23)	15
	*Quick*DASH	32 (26)	33	23 (23)	16

The scores for both scales range from 0 (no disability) to 100 (most severe disability)

**Table 3:** The area under the receiver operating characteristic (ROC) curve for the DASH and the *Quick*DASH, constructed using the change scores for patients classified, according to their response to the global item about self-rated improvement in arm status after surgery, into "much better", "somewhat better" or "unchanged"; item administered to 82 participants.

	**Area under ROC curve (95% CI)**			
	"better" vs "unchanged"		"much better" vs "somewhat better"	
	(n = 65)*	(n = 9)	(n = 47)	(n = 18)
	
DASH	0.81 (0.70–0.89)		0.71 (0.59–0.82)	
*Quick*DASH	0.82 (0.71–0.90)		0.68 (0.56–0.79)	
difference between DASH and *Quick*DASH	0.01 (0.05–0.07), p = 0.8		0.03 (-0.03–0.09), p = 0.3	

*combining patients responding "much better" and those responding "somewhat better" to the global item.

The remaining responders to the global item were 6 with "much worse" and 2 "somewhat worse" responses.

**Table 4:** Reliability

**Scale**	**Test-retest reliability**		**Cross-sectional reliability**				**Agreement with DASH**			
	**Baseline**		**Baseline**		**Follow-up**		**Baseline**		**Follow-up**	
	Agreement	Difference*					Agreement	Difference	Agreement	Difference
	ICC (95% CI)^†^	mean (95% CI)	alpha	ITC range	alpha	ITC range	ICC (95% CI)^†^	mean (95% CI)^‡^	ICC (95% CI)^†^	mean (95% CI)^‡^
**DASH**	0.93 (0.86–0.97)	0.9 (- 2.3–4.0)^a^	0.97	0.41–0.86	0.98	0.57–0.90	1	0	1	0
***Quick*** **DASH**	0.93 (0.87–0.97)	-0.2(-3.6–3.1)^b^	0.92	0.42–0.83	0.95	0.63–0.88	0.96 (0.84–0.98)	4.2 (3.2–5.3)	0.97 (0.92–0.99)	2.6 (1.7–3.4)
**Random-11 1**	0.92 (0.84–0.96)	-2.1(-5.7–1.5)^c^	0.93	0.55–0.82	0.95	0.55–0.88	0.96 (0.92–0.98)	3.1 (2.0–4.2)	0.97 (0.91–0.98)	3.4 (2.4–4.5)
**Random-11 2**	0.93 (0.85–0.96)	-0.3(-3.7–3.1)^b^	0.91	0.42–0.81	0.95	0.62–0.87	0.97 (0.96–0.98)	1.7 (0.7–2.7)	0.99 (0.98–0.99)	0.7 (0–1.4)

*calculated as the score at time 1 minus the score at time 2.

^†^All ICC values were statistically significant (p < 0.001).

^‡^All differences between the DASH and the other 3 forms were significant (p ≤ 0.001) except for random-11 form 2 at follow-up (p = 0.053)

^a^p = 0.6, ^b^p = 0.9, ^c^p = 0.2.

ICC, intraclass correlation coefficient; alpha, Cronbach alpha coefficient; ITC, item-total correlation

## References

[B1] Hudak PL, Amadio PC, Bombardier C (1996). Development of an upper extremity outcome measure: the DASH (disabilities of the arm, shoulder and hand) [corrected]. The Upper Extremity Collaborative Group (UECG). Am J Ind Med.

[B2] Davis AM, Beaton DE, Hudak P, Amadio P, Bombardier C, Cole D, Hawker G, Katz JN, Makela M, Marx RG, Punnett L, Wright JG (1999). Measuring disability of the upper extremity: a rationale supporting the use of a regional outcome measure. J Hand Ther.

[B3] Beaton DE, Katz JN, Fossel AH, Wright JG, Tarasuk V, Bombardier C (2001). Measuring the whole or the parts? Validity, reliability, and responsiveness of the disabilities of the arm, shoulder and hand outcome measure in different regions of the upper extremity. J Hand Ther.

[B4] The DASH outcome measure. Institute for Work & Health, Toronto, Canada. http://www.dash.iwh.on.ca.

[B5] Beaton DE, Wright JG, Katz JN (2005). Development of the QuickDASH: comparison of three item-reduction approaches. J Bone Joint Surg Am.

[B6] Streiner DL, Norman GR (1995). Health Measurement Scales A practical guide to their development and use.

[B7] Norman G (2003). Hi! How are you? Response shift, implicit theories and differing epistemologies. Qual Life Res.

[B8] Liang MH (2000). Longitudinal construct validity: establishment of clinical meaning in patient evaluative instruments. Med Care.

[B9] Terwee CB, Dekker FW, Wiersinga WM, Prummel MF, Bossuyt PM (2003). On assessing responsiveness of health-related quality of life instruments: guidelines for instrument evaluation. Qual Life Res.

[B10] Gummesson C, Atroshi I, Ekdahl C (2003). The disabilities of the arm, shoulder and hand (DASH) outcome questionnaire: longitudinal construct validity and measuring self-rated health change after surgery. BMC Musculoskelet Disord.

[B11] Altman DG, Bland JM (1994). Diagnostic tests 3: receiver operating characteristic plots. BMJ.

[B12] Zweig MH, Campbell G (1993). Receiver-operating characteristic (ROC) plots: a fundamental evaluation tool in clinical medicine. Clin Chem.

[B13] Schuck P (2004). Assessing reproducibility for interval data in health-related quality of life questionnaires: which coefficient should be used?. Qual Life Res.

[B14] Atroshi I, Gummesson C, Andersson B, Dahlgren E, Johansson A (2000). The disabilities of the arm, shoulder and hand (DASH) outcome questionnaire: reliability and validity of the Swedish version evaluated in 176 patients. Acta Orthop Scand.

[B15] Gummesson C, Atroshi I, Ekdahl C (2003). Performance of health-status scales when used selectively or within multi-scale questionnaire. BMC Med Res Methodol.

